# Effects of *Lacticaseibacillus rhamnosus* L156.4 and *Lactococcus lactis* NCDO 2118 strains on reducing alcohol intake and preference in an animal model of high alcohol consumption and preference

**DOI:** 10.1038/s41598-026-48677-y

**Published:** 2026-04-14

**Authors:** Mariana Siqueira Amormino, Renato Elias Moreira-Júnior, Rafaella Antunes Vasconcelos, Isabela Nascimento dos Santos, Ana Beatriz Ramos de Moura Gonçalves, Mírian Velten Mendes, Julia de Lima Santos, Fernanda Alvarenga Lima Barroso, Vasco Ariston de Carvalho Azevedo, Tatiani Uceli Maioli, Ana Maria Caetano Faria, Enio Ferreira, Ana Lúcia Brunialti-Godard

**Affiliations:** 1 Laboratório de Genética Animal e Humana, Departamento de Genética, Ecologia e Evolução, Instituto de Ciências Biológicas, Universidade Federal de Minas Gerais, Belo Horizonte, Brazil; 2 Laboratório de Genética Celular e Molecular, Departamento de Genética, Ecologia e Evolução, Instituto de Ciências Biológicas, Universidade Federal de Minas Gerais, Belo Horizonte, Brazil; 3 Laboratório de Imunobiologia, Departamento de Bioquímica e Imunologia, Instituto de Ciências Biológicas, Universidade Federal de Minas Gerais, Belo Horizonte, Brazil; 4 Laboratório de Comportamento Celular, Departamento de Patologia, Instituto de Ciências Biológicas, Universidade Federal de Minas Gerais, Belo Horizonte, Brazil

**Keywords:** Probiotic, *Lactococcus lactis* NCDO 2118, *Lacticaseibacillus rhamnosus* L156.4, Alcohol use disorder, Reward system, Neuroinflammation, Dut-brain axis, Microbiology, Neuroscience

## Abstract

Alcohol use disorder (AUD) involves a progressive series of neuroadaptations influenced by interactions along the gut-brain axis. Disruption of the intestinal barrier enables bacterial-derived metabolites and inflammatory mediators to translocate into the systemic circulation, amplifying neuroimmune activation within mesocorticolimbic reward circuits. Probiotic strains have emerged as promising adjuvant therapies for AUD due to their ability to regulate the gut microbiota and thereby influence neurotransmission. In this study, we investigated the potential of a probiotic blend containing the strains *Lacticaseibacillus rhamnosus* L156.4 and *Lactococcus lactis* NCDO 2118 to reduce alcohol intake and preference behaviors in a free-access drinking paradigm (H₂O vs. 10% v/v ethanol). The blend significantly decreased ethanol intake and preference and was accompanied by striatum upregulation of genes related to dopaminergic inhibitory control and downregulation of inflammation-related genes. Serum biochemical parameters remained unchanged and comparable between vehicle- and probiotic-treated groups, supporting the safety of the intervention. Additionally, we observed improved tissue integrity and structural restoration in both the colon and liver. Behavioral assessments using the open field test and marble-burying tests revealed reduced anxiety- and compulsive-like behaviors in the treated groups. These results suggest that this probiotic blend modulates distinct pathways, thereby attenuating alcohol`s rewarding effects and associated compulsive behaviors.

**Supplementary Information:**

The online version contains supplementary material available at 10.1038/s41598-026-48677-y.

## Introduction

Alcohol use disorder (AUD) is a complex condition characterized by dysregulation of neural and immune circuits and remains a significant public health concern, contributing to more than three million deaths globally annually^1^. The rewarding effects of ethanol arise from ethanol-induced remodeling of cortico-striatal circuits, involving a loss of GABAergic inhibitory control together with increased dopaminergic and glutamatergic drive, particularly in the prefrontal cortex and striatum^2^. This disruption of the excitatory–inhibitory balance within striatal networks modulates the transcriptional regulation of membrane receptors that govern synaptic plasticity, thereby reinforcing addictive behaviors^2,3^. In this context, the leucine-rich repeat kinase 2 (*Lrrk2*) gene, previously identified in our studies as a modulator of ethanol responses^2,3^, has been implicated in the regulation of dopamine receptor distribution and signaling in the striatum^4^.

The assessment of alcohol preference behavior reflects the relationship between the reward experienced within a defined time window and the subsequent motivation to voluntarily seek that stimulus^5^. Studies from our group demonstrate that the abrupt withdrawal of hedonic stimuli, such as high-fat diets, promotes a behavioral shift from compulsive eating to alcohol intake^5,6^. Moreover, the overlap between the reinforcement produced by these stimuli suggests that a primary, diet-induced dysbiosis may condition compulsive behavior and increase vulnerability to alcohol use disorder. In this sense, diets rich in fats and sugars can promote microbiota alterations that create a vulnerable environment for high ethanol consumption, by enrichment of carbohydrate-metabolizing bacterial phyla associated with low-grade inflammation and metabolic dysfunction^7^.

Beyond its central nervous system effects, ethanol intake perturbs the gut microbiota, a key regulator of neurobehavioral homeostasis via the gut-brain axis^8^. Ethanol consumption consistently reduces the abundance of beneficial bacteria such as *Lacticaseibacillus*^9^, which have demonstrated potential in mitigating AUD due to their capacity to modulate brain dopaminergic activity and, in turn, reduce voluntary ethanol intake and related behaviors^9,10^. Additionally, *Lactococcus lactis* has demonstrated neuromodulatory potential in murine models through gamma-aminobutyric acid (GABA) production, exerting anxiolytic effects by modulating neural activity^11,12^. Elevated GABA levels appear to reduce dopamine release and inhibit pro-inflammatory cytokine secretion, thereby influencing synaptic plasticity and behavioral outcomes.

The potential of *Lactococcus lactis* NCDO 2118 in managing AUD is attributed to its role as a neuromodulator and metabolic regulator that supports brain function via the gut-brain axis^13–15^, with ethanol exposure appearing particularly relevant to the activity of this bacterial strain^13–16^. Some strains of *Lacticaseibacillus rhamnosus* display enhanced acetaldehyde metabolism^17,18^, suggesting a complementary interaction with *Lactococcus lactis* NCDO 2118 that may promote hepatic alcohol detoxification and reduce intestinal inflammation^19^.

Given that their metabolites help maintain bidirectional functional communication between the intestine and the central nervous system and modulate GABAergic and dopaminergic signaling, thereby influencing behavioral responses mediated by these pathways, we hypothesized that a blend of L. rhamnosus L156.4 and L. lactis NCDO 2118 could reduce ethanol intake and preference^19–21^. To test this, conventional C57BL/6 mice were fed a High Sugar and Butter diet (HSB) for eight weeks (T1) to induce binge-eating behaviors associated with reward pathways^22^. Subsequently, the HSB diet was replaced with standard chow, and ethanol was introduced for two weeks in a two-bottle free choice paradigm (T2). Finally, the animals were administered a 1:1 probiotic blend of *Lacticaseibacillus rhamnosus* L156.4 and *Lactococcus lactis* NCDO 2118 (T3).

## Results

### Body weight and biochemical parameters remain unaffected by probiotic administration

**Fig. 1:**
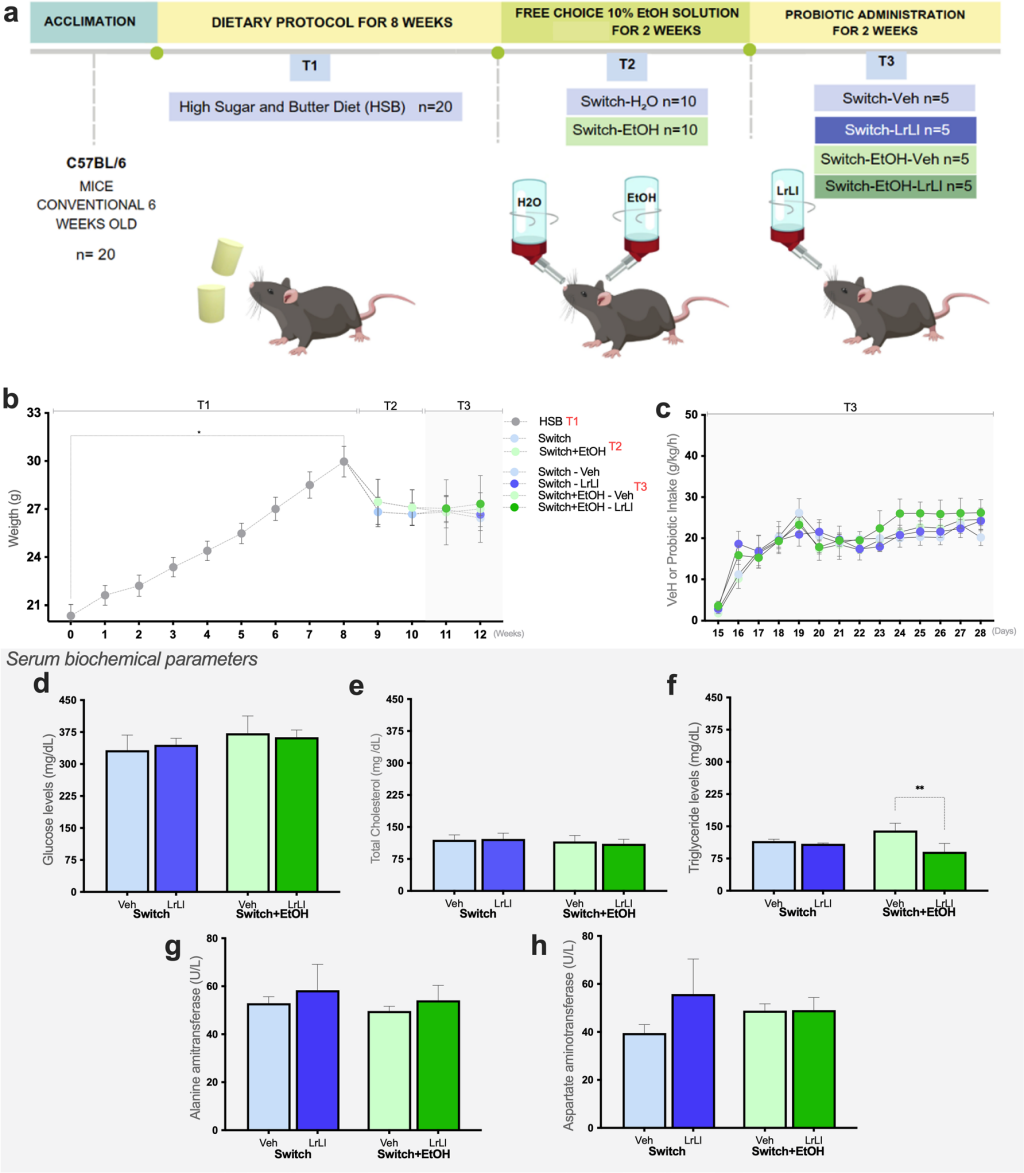
Probiotic administration does not affect body weight or most biochemical parameters, except triglycerides. (**a**) Experimental timeline showing HSB exposure (T1), the ethanol free-choice period during T2 (free access to H₂O vs. 10% v/v ethanol for +EtOH groups), and the intervention during T3. Switch (non-ethanol) animals remained with a single H₂O bottle during T2 and T3. During T3, vehicle or the LrLl blend was provided daily for 3.5 h (2:00–5:30 PM) as the sole liquid source, with solid diet available ad libitum. (**b**) Mean weekly body weight (**g**). (**c**) Vehicle or probiotic intake (g/kg/h). Serum levels of (**d**) Glucose (mg/dL), (**e**) Total cholesterol (mg/dL), (**f**) Triglycerides (mg/dL), (**g**) Alanine aminotransferase (U/L), and (**h**) Aspartate aminotransferase (U/L). Statistical analysis: (**b**–**c**) Two-way repeated measures ANOVA followed by Sidak’s post hoc test; (**d**–**h**) Kruskal–Wallis test. (*p < 0.05, **p < 0.01)

The experimental timeline is summarized in Fig. 1a, including the HSB exposure phase (T1), the ethanol free-choice period during T2, and the probiotic intervention during T3. In T2, ethanol-exposed animals had free access to two drinking bottles (H₂O vs. 10% v/v ethanol), whereas Switch animals remained with a single H₂O bottle. During T3, vehicle or the LrLl probiotic blend was provided daily for 3.5 h (2:00–5:30 PM) as the sole liquid source, with solid diet available ad libitum (Fig. 1a). No differences (*p* > 0.05) in body weight were observed between the groups throughout the experiment (Fig. 1b). The average body weights at the end of the experiment (T3) were as follows: Switch-Veh = 25.72 ± 2.10 g, Switch-LrLl = 26.66 ± 1.69 g, Switch+EtOH-Veh = 27.01 ± 4.65 g, and Switch+EtOH-LrLl = 27.32 ± 1.58 g. Probiotic or vehicle intake (Fig. 1c), as well as serum glucose (Fig. 1d) and total cholesterol (Fig. 1e) levels were similar across the groups. However, triglyceride levels were significantly lower in the Switch+EtOH-LrLl group than in the Switch+EtOH-Veh (*p* = 0.0076, Fig. 1e), while no difference was detected between Switch+EtOH-Veh and Switch-Veh (*p* > 0.05). The levels of alanine transaminase (Fig. 1g) and aspartate transaminase (Fig. 1h) did not differ between the groups.

### Probiotic blend reduces ethanol intake and preference

**Fig. 2:**
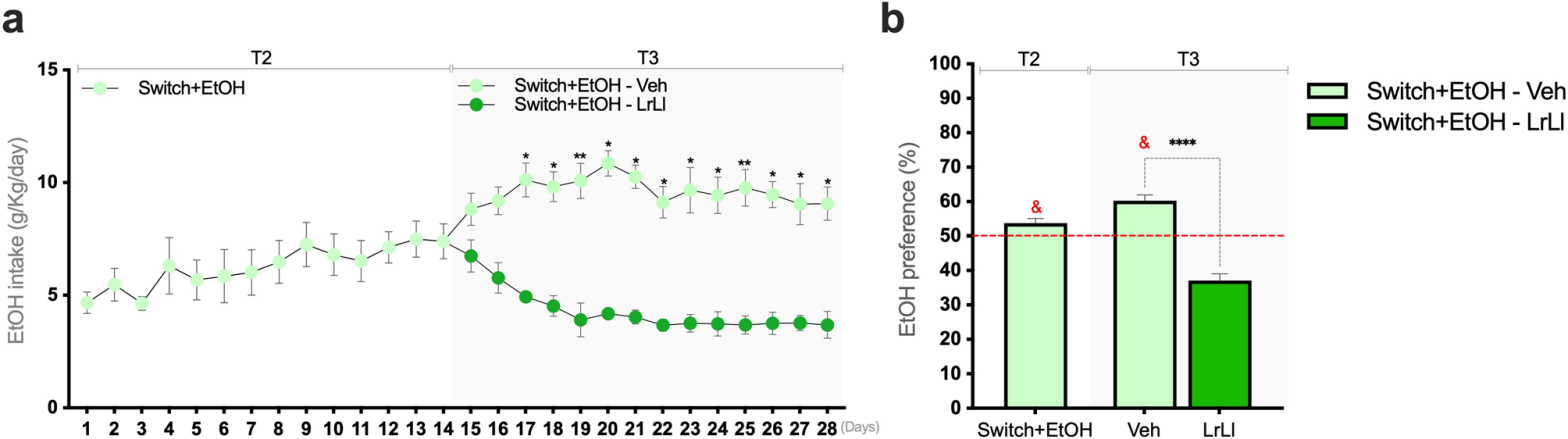
The probiotic blend reduced ethanol consumption and preference. (**a**) Daily pure ethanol intake (g/kg/day) at T2 and T3, calculated from the gravimetrically measured consumption of the 10% (v/v) ethanol solution and normalized to body weight. (**b**) Ethanol preference (%) at T2 and T3. Statistical analysis: (**a**) Two-way repeated measures ANOVA followed by Sidak’s post hoc test. (**b**) One-sample t-test comparing ethanol preference to the hypothetical value of 50.1% (indicated by the red dashed line with statistical differences denoted by &); unpaired t-test between groups at T3 (Veh vs. LrLl). (*p, & < 0.05; **p < 0.01; < ****p < 0.0001). In this experiment, one day corresponded to 20.5 h of access.

During phase T3, mice in the Switch+EtOH-LrLl group showed a progressive decline in voluntary ethanol intake (Fig. 2a). Values shown in Fig. 2a represent pure ethanol intake (g/kg/day), calculated from the gravimetrically measured consumption of the 10% (v/v) ethanol solution and normalized to body weight. At T2, ethanol preference in Switch+EtOH animals exceeded the 50.1% reference value (*p* = 0.0184; Fig. 2b, T2). This elevated preference persisted in the vehicle-treated group at T3 (*p* = 0.0037), whereas the Switch+EtOH-LrLl group showed a marked reduction (*p* < 0.0001; Fig. 2b, T3), corresponding to a 43.97% decrease at the end of T3. Water intake is shown in Supplementary Fig. S1. Statistical comparison between the last day of T2 and the first day of T3 showed no significant differences between these time points (Switch+EtOH-Veh, *p* = 0.1419; Switch+EtOH-LrLl, *p* > 0.9999); these results are presented in Supplementary Fig. S2. Gravimetric intake of the 10% (v/v) ethanol solution, expressed as g/kg/day and mL/kg/day, as well as daily ethanol preference trajectories during T2 and T3, are presented in Supplementary Fig. S3.

### The probiotic blend restores intestinal mucosal integrity

**Fig. 3:**
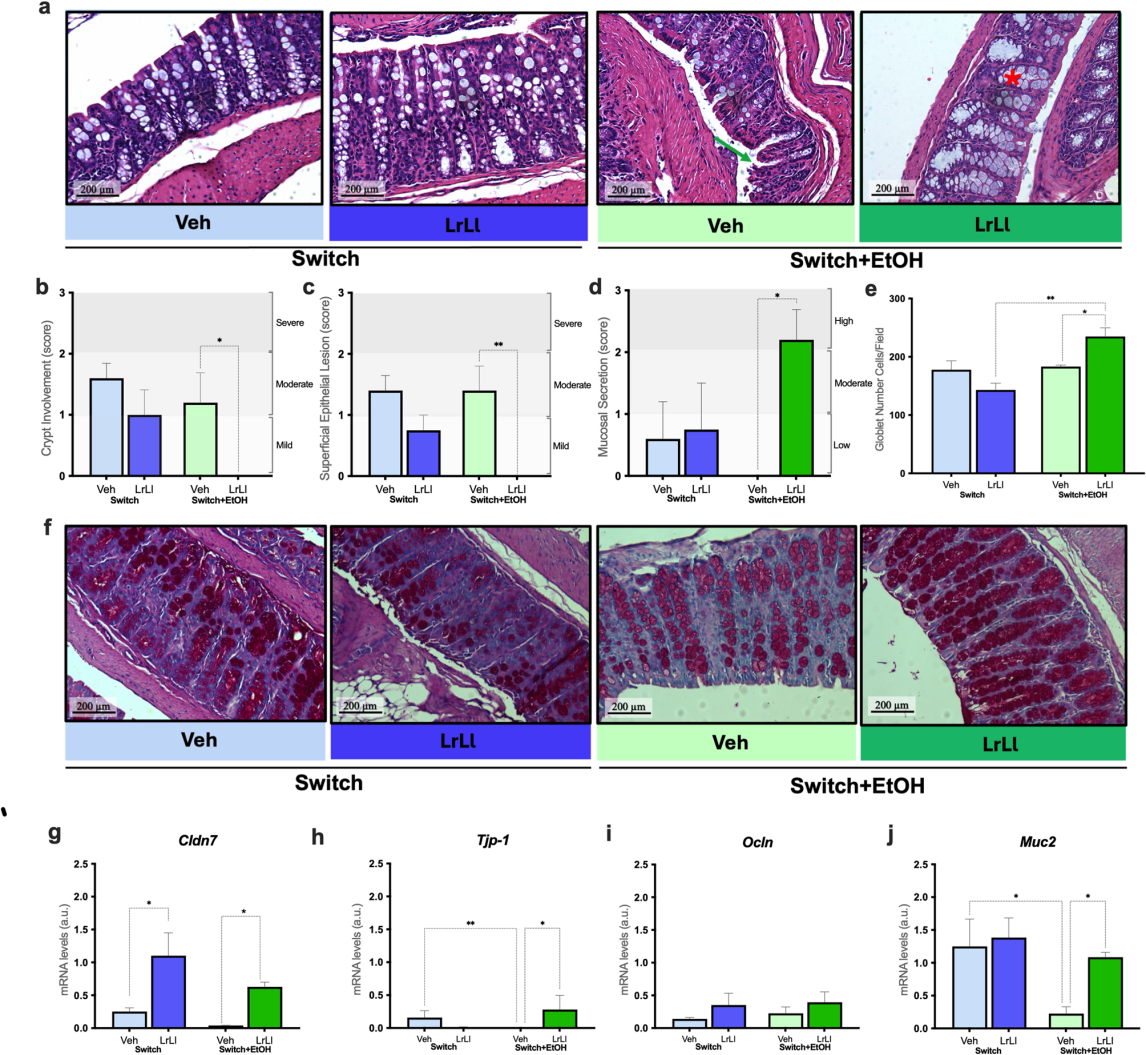
The probiotic blend restores intestinal mucosal integrity. (**a**) Representative H&E-stained colon sections. Score analysis of (**b**) crypt involvement, (**c**) superficial epithelial lesions, and (**d**) mucosal secretion. (**e**) Goblet cells count per field. (**f**) Representative PAS-stained colon sections. Transcriptional regulation of (g) *Cldn7*, (**h**) *Tjp1*, (**i**) *Ocln*, and (**j**) *Muc2*. Statistical analysis: (b–e, g–j) Kruskal–Wallis test. (*p < 0.05, **p < 0.01). Histological quadrants and graphs are represented in light blue and dark blue, indicating the Switch-Veh and Switch-LrLl groups, respectively. Switch+EtOH-Veh and Switch+EtOH-LrLl groups are shown in light green and dark green, respectively. In (**a**), the green arrow indicates epithelial disruption, and the red asterisk indicates a mucus/goblet cell–rich area.

To verify alterations in the colon mucosa, histological analysis was performed in HE- stained tissues (Fig. 3a) and the results revealed that tissues from the Switch groups, showed no evident tissue alterations. Mucosal damage was observed in Switch+EtOH-Veh animals, including epithelial disruption and crypt deformation, when compared to Switch-Veh. In contrast, Switch+EtOH-LrLl animals showed preserved mucosal architecture and visible mucus production. Representative features are indicated in Fig. 3a (green arrow, epithelial disruption; red asterisk, mucus/goblet cell-rich area). Integrity scores revealed increased crypt damage (*p* = 0.0202) and more epithelial lesions (*p* = 0.0018) in the Veh group than in the LrLl group (Figs. 3b-c). Mucus content was also higher in Switch+EtOH-LrLl animals (*p* = 0.0072; Fig. 3d). PAS staining revealed a greater number of goblet cells in Switch+EtOH-LrLl compared to Switch+EtOH-Veh (*p* = 0.0087) and Switch-Veh (*p* = 0.0001) (Fig. 3e), a finding confirmed by higher density of magenta-stained cells in histological sessions (Fig. 3f). Morphological changes were corroborated by transcriptional regulation of genes tight junction genes, with *Tjp1* and *Cldn7* upregulated in Switch+EtOH-LrLl animals (Fig. 3g and h), wheareas *Ocln* transcription remained unchanged (Fig. 3i). Consistent with mucus restoration, *Muc2* transcriptional levels were significantly elevated in Switch+EtOH-LrLl compared to Switch+EtOH-Veh (Fig. 3j).

### Probiotic strains persist in the intestine and partially integrate with the resident microbiota

**Fig. 4:**
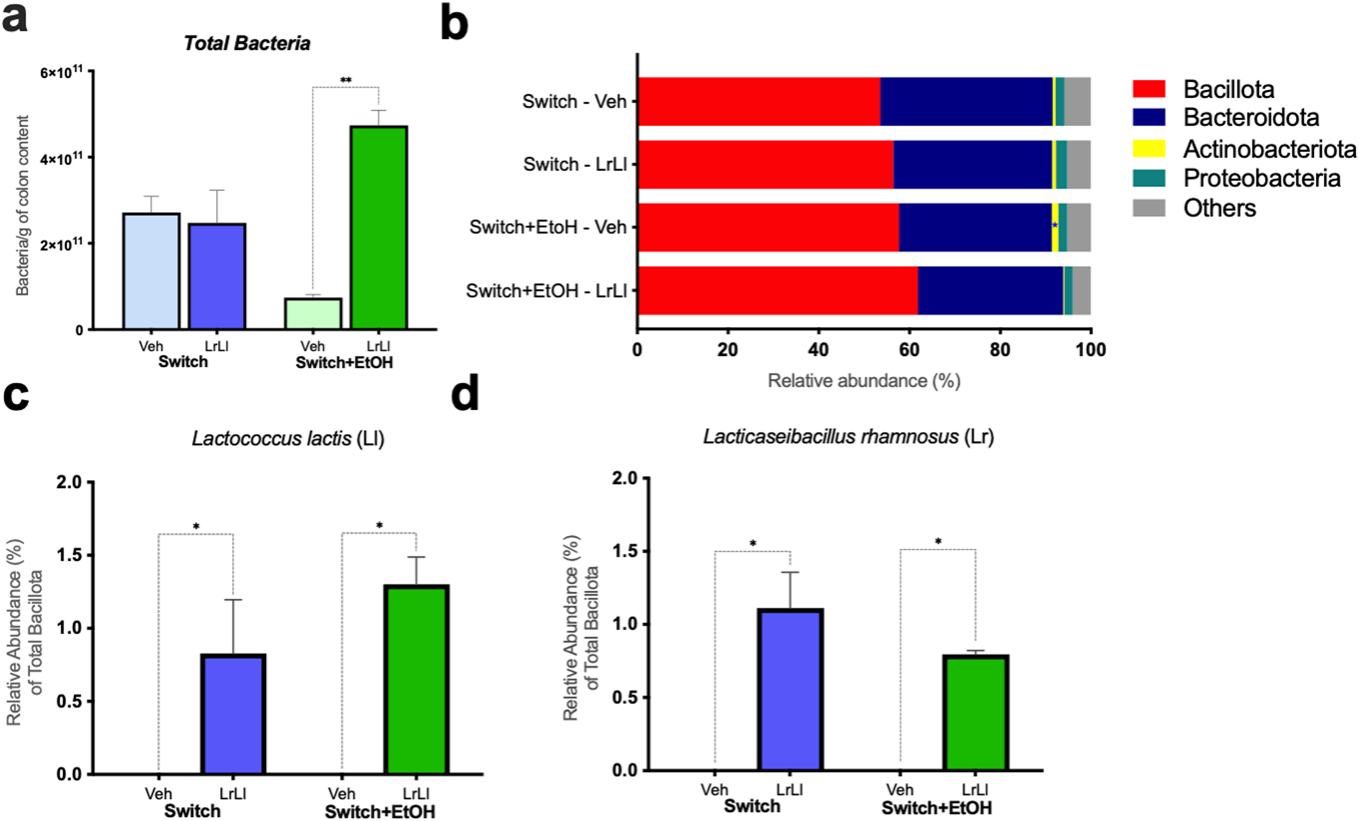
Probiotic strains colonize the gut microbiota following administration of the blend. (**a**) Total bacterial load (bacteria/g of colonic content) determined by absolute 16 S rRNA qPCR. (**b**) Relative abundance of bacterial phyla (%). (**c**) Relative abundance of *Lactococcus lactis*. (**d**) Relative abundance of *Lacticaseibacillus rhamnosus*. Statistical analysis: (**a**–**d**) Kruskal–Wallis test. *p < 0.05, **p < 0.01. Graphs are represented in light blue and dark blue, indicating the Switch + H₂O-Veh and Switch + H₂O-LrLl groups, respectively. Switch+EtOH-Veh and Switch+EtOH-LrLl groups are shown in light green and dark green, respectively.

Quantitative analysis of colonic content using 16 S rRNA gene-targeted qPCR revealed a significant increase in total bacterial load in the Switch+EtOH-LrLl compared to Switch+EtOH-Veh (Fig. 4a). At the phylum level, no significant differences were observed in Bacillota, Bacteroidota, or Proteobacteria (Fig. 4b). However, the Actinomycetota relative abundance was elevated (*p* = 0.0259) in the Switch+EtOH-Veh group compared to Switch-Veh animals. The relative abundance of *Lacticaseibacillus rhamnosus* L156.4 and *Lactococcus lactis* NCDO 2118 was significantly increased in the groups treated with the probiotic blend, confirming the successful partial integration of these strains in the gut microbiota following administration (Fig. 4c and d). The probiotic strains’ tolerance to ethanol was assessed across escalating concentrations as shown in Supplementary Fig. S5.

### Probiotic blend attenuates ethanol-associated hepatic steatosis and inflammation

**Fig. 5:**
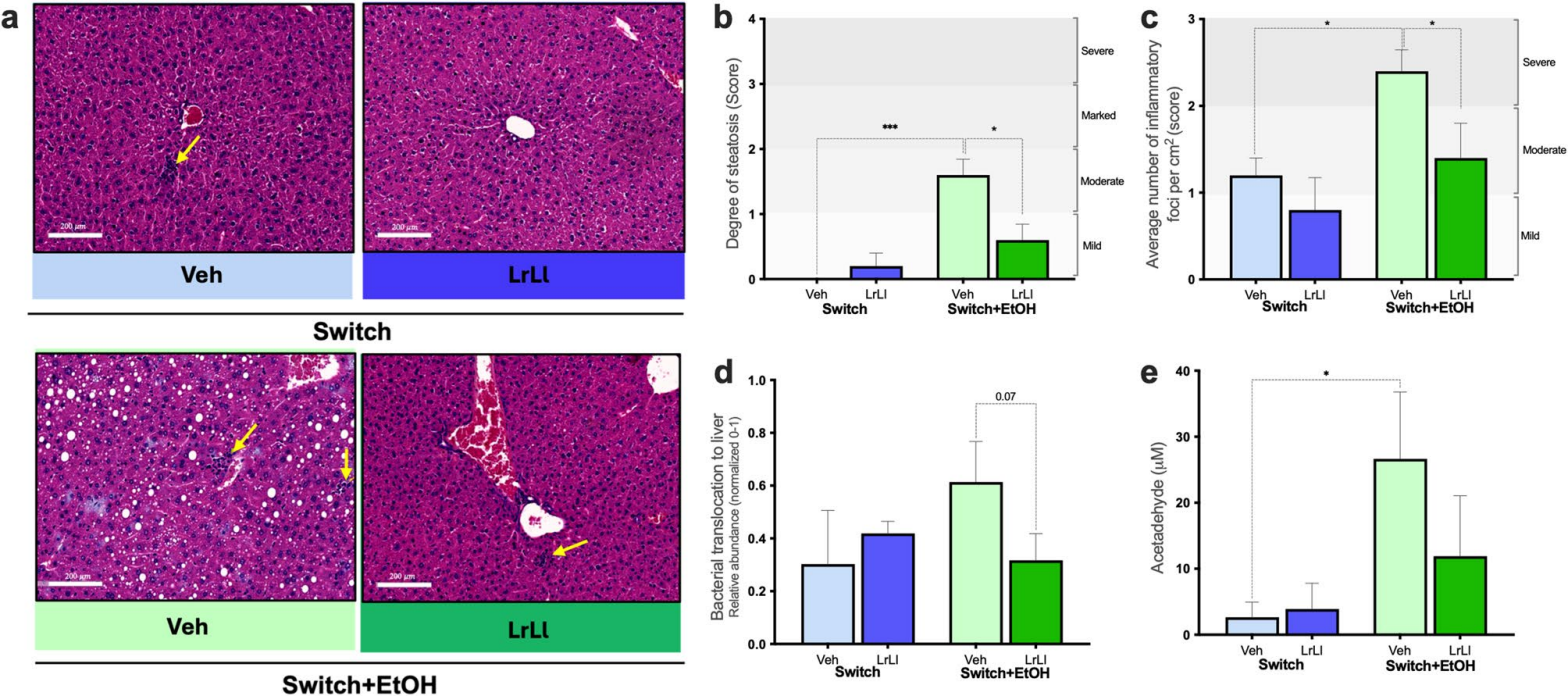
Probiotic blend attenuates ethanol-induced hepatic steatosis and inflammation. (**a**) Representative HE-stained liver sections. (**b**) Average number of inflammatory foci per cm² (score; inflammatory foci were defined as clusters of infiltrating mononuclear cells). (**c**) Degree of steatosis (score). (**d**) Bacterial translocation to the liver based on total bacterial DNA quantification. (**e**) Serum acetaldehyde concentration (µM). Statistical analysis: (**b**–**d**) Kruskal–Wallis test; (**e**) ordinary two-way ANOVA. (*p < 0.05; **p < 0.01; ***p < 0.001). Histological quadrants and graphs are represented in light blue and dark blue, indicating the Switch-Veh and Switch-LrLl groups, respectively. Switch+EtOH-Veh and Switch+EtOH-LrLl groups are shown in light green and dark green, respectively.

Morphological analysis of HE-stained liver sections revealed a cluster of inflammatory cells in the liver parenchyma of Switch+EtOH-Veh (Fig. 5a). These animals also exhibited lipid vacuoles indicative of moderate hepatic steatosis (Fig. 5b), and a high density of inflammatory foci that yielded severe histopathology scores (*p* < 0.05, Fig. 5c) relative to both Switch-Veh and Switch+EtOH-LrLl groups. Bacterial translocation, assessed by quantifying total bacterial DNA in liver tissue, remained low in the Switch+EtOH-LrLl group, showing levels comparable to those observed in the Switch-Veh group (Fig. 5d). Consistent with their greater ethanol intake, serum acetaldehyde concentrations were significantly elevated in the Switch+EtOH-Veh group than in the other groups (*p* = 0.0433, Fig. 5e). Within ethanol-exposed groups, correlation analyses further supported this relationship, revealing positive trend associations between individual ethanol intake and serum acetaldehyde levels (Supplementary Fig. S4).

### Probiotic blend administration modulates animal behavior

**Fig. 6:**
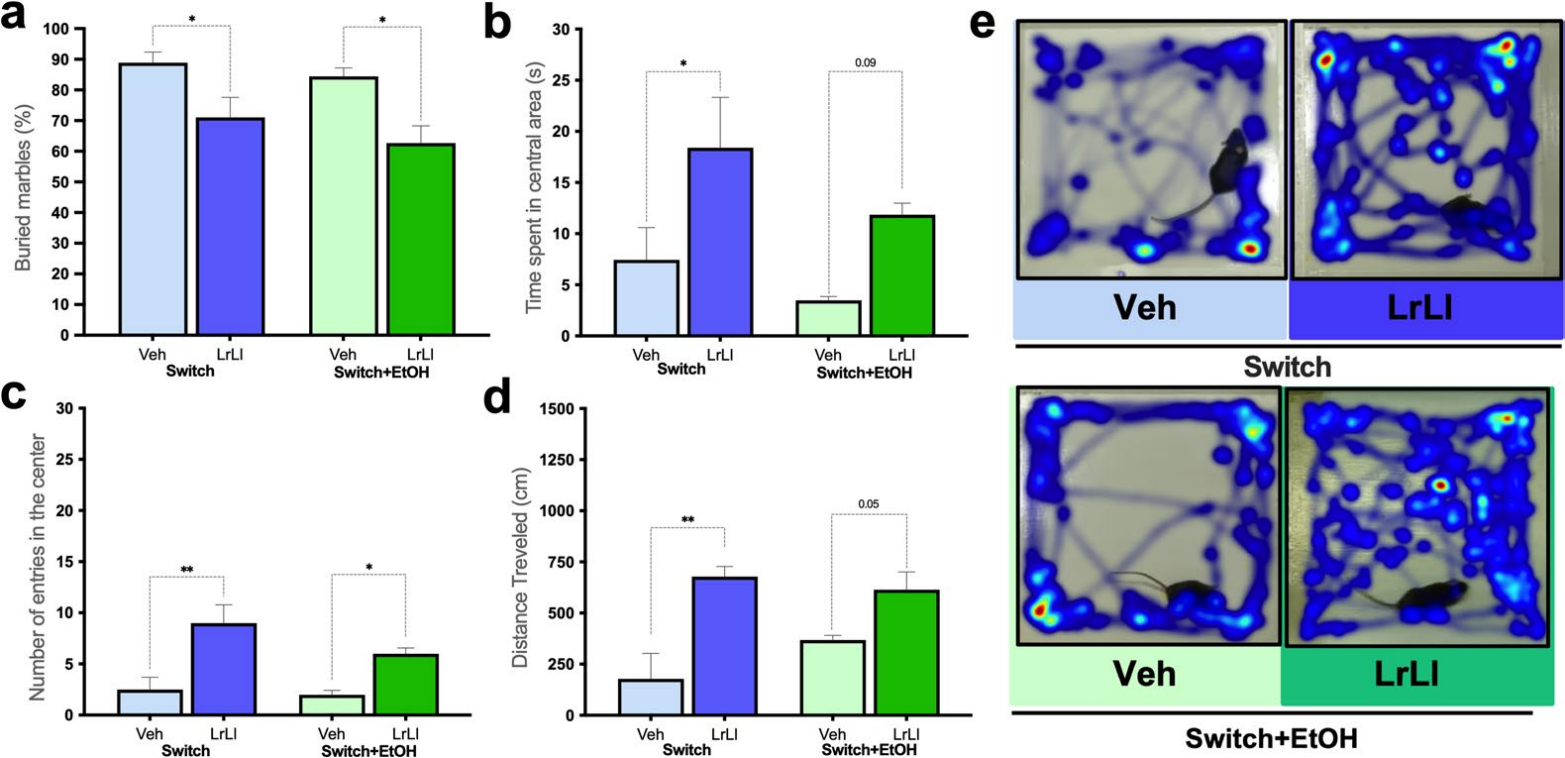
Probiotic Blend Reduces Compulsive-like and Anxiety-related Behaviors in Mice. (**a**) Percentage of buried marbles in the marble burying test. In the open field test: (**b**) Time spent in the central area (s), (**c**) Number of entries into the central area, (**d**) Distance traveled (cm), and (**e**) Representative images of exploratory behavior. Statistical analysis: (**a**–**d**) Kruskal–Wallis test. (*p < 0.05, **p < 0.01). Graphs and exploratory behavior quadrants are represented in light blue and dark blue, indicating the Switch-Veh and Switch-LrLl groups, respectively. Switch+EtOH-Veh and Switch+EtOH-LrLl groups are shown in light green and dark green, respectively.

Animals treated with the probiotic blend (Switch-LrLl and Switch+EtOH-LrLl) exhibited reduced (*p* < 0.05) compulsive-like behavior in the Marble Burying test compared to their respective vehicle treated controls (Fig. 6a). In the Open Field Test, both the Switch-Veh and Switch+EtOH-Veh groups displayed a significantly (*p* < 0.05) shorter latency to first entry (Fig. 6b), reduced time spent in the central area (Fig. 6c), and decreased total distance traveled (Fig. 6d), when compared to their respective probiotic-treated groups (Switch-LrLl and Switch+EtOH-LrLl). These findings indicate that probiotic administration normalized the natural exploratory behavior of mice (Fig. 6e).

### Probiotic blend modulates striatal gene profiles in ethanol-exposed mice

**Fig. 7:**
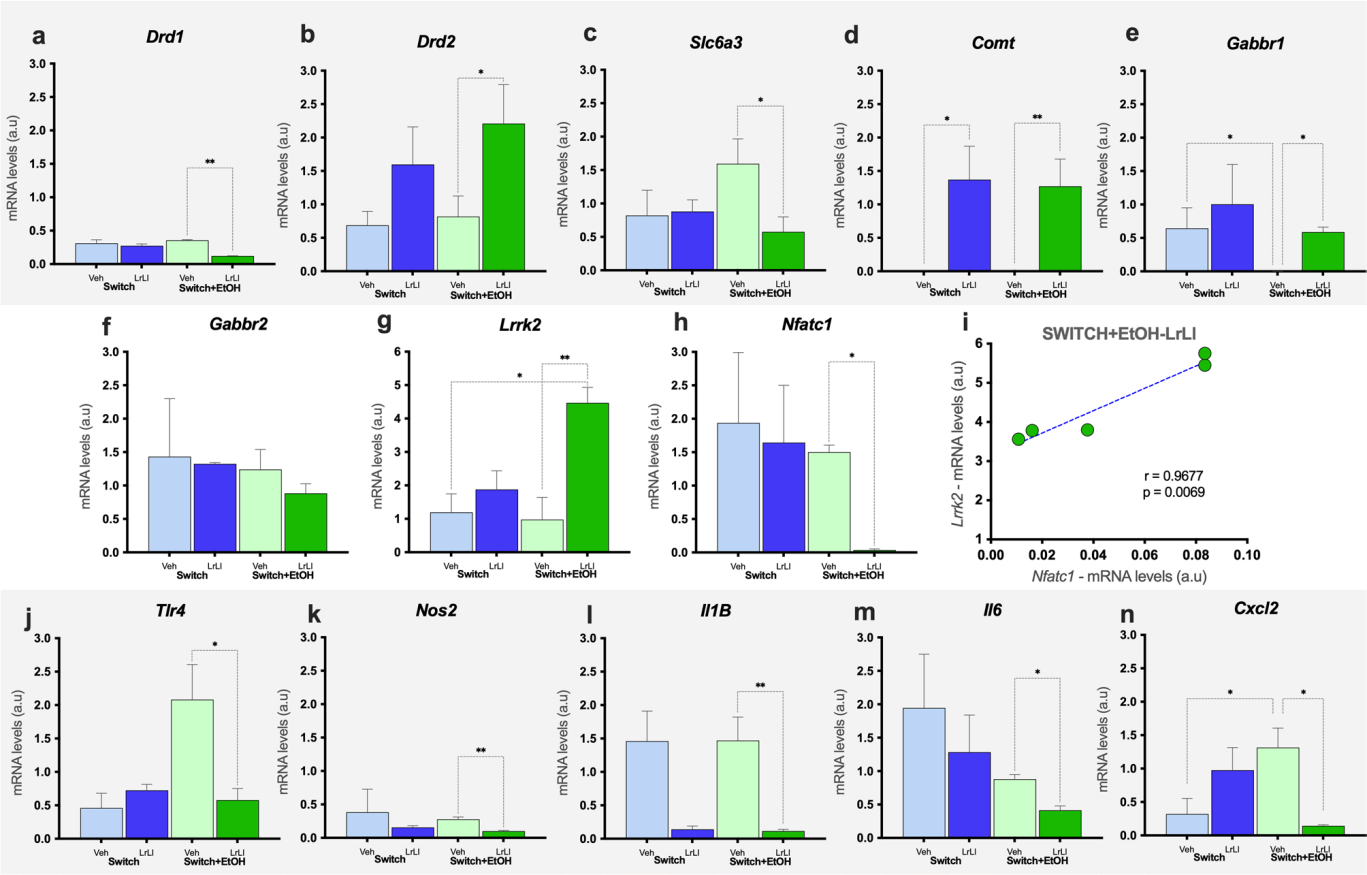
Experimental treatments modulate striatal mRNA levels. Transcriptional regulation of: (**a**) *Drd1*, (**b**) *Drd2*, (**c**) *Slc6a3*, (**d**) *Comt*, (**e**) *Gabbr1*, (**f**) *Gabbr2*, (**g**) *Lrrk2*, (**h**) *Nfatc1*, (**i**) correlation between *Lrrk2* and *Nfatc1* transcript levels, (**j**) *Tlr4*, (**k**) *Nos2*, (**l**) *Il1β*, (**m**) *Il6*, (**n**) *Cxcl2*. Statistical analysis: (**a**-**h**, **j**-**n**) Kruskal–Wallis test for group; (**i**) Pearson’s correlation with simple linear regression. (*p < 0.05, **p < 0.01). Graphs are represented in light blue and dark blue, indicating the Switch-Veh and Switch-LrLl groups, respectively. Switch+EtOH-Veh and Switch+EtOH-LrLl groups are shown in light green and dark green, respectively.

Transcriptional regulation of dopaminergic and GABAergic signaling genes in the striatum revealed a pattern of differentially regulated genes across the experimental groups. Among dopaminergic markers, *Drd1* was significantly downregulated in Switch+EtOH-LrLl mice relative to Switch+EtOH-Veh (*p* = 0.0014, Fig. 7a), while *Drd2* was upregulated (*p* = 0.0371, Fig. 7b). The dopamine transporter *Slc6a3* exhibited higher transcript levels in Switch+EtOH-Veh than in Switch+EtOH-LrLl animals (*p* = 0.0382, Fig. 7c). The catabolic enzyme gene *Comt* was upregulated in LrLl-treated groups (*p* < 0.05, Fig. 7d). Among GABAergic markers, *Gabbr1* transcripts differed significantly between vehicle and LrLl-treated groups (*p* = 0.0096, Fig. 7e), whereas *Gabbr2* remained unchanged (Fig. 7f). Additionally, the *Lrrk2* gene showed elevated transcription (*p* = 0.0024, Fig. 7g) in Switch+EtOH-LrLl mice compared with Switch+EtOH-Veh and Switch-Veh, while the Nfatc1 gene was downregulated (*p* = 0.0425, Fig. 7h) in Switch+EtOH-LrLl. *Lrrk2* and *Nfatc1 mRNA* levels were correlated (*r* = 0.968, *p* = 0.0069; simple linear regression, *y* = 28.52* x* + 3.149) (Fig. 7i). *Tlr4*, *Nos2*, *Il1β* and *Il6* genes were upregulated only in the Switch+EtOH-Veh group (*p* < 0.05, Fig. 7j-m). *Cxcl2* followed a similar pattern and was additionally downregulated in Switch-Veh compared to Switch+EtOH-Veh (*p* = 0.0453, Fig. 7n).

## Discussion

This study uncovers a novel probiotic approach for reducing alcohol intake and preference, mediated by the reestablishment of intestinal integrity and modulation of neuroinflammatory responses via the gut-brain axis. Hedonic seeking of ethanol was observed following withdrawal from a diet rich in sugar and fat, indicating a robust ethanol preference behavior^5^. We demonstrated that a two weeks of treatment with a blend containing *Lacticaseibacillus rhamnosus* L156.4 and *Lactococcus lactis* NCDO 2118 significantly reduced ethanol consumption and preference. This reduction coincided with a higher abundance of these strains in the colon of treated animals, and we verified their ethanol tolerance in the present study, underscoring the gut microbiota’s capacity to modulate alcohol-seeking behavior. During T3, the Switch+EtOH-LrLl group showed a 59.37% decrease in voluntary ethanol intake, accompanied by a 43.97% linear decline in preference, which persisted until the end of the experiment. Serum biochemical analyses demonstrated the safety of the probiotic intervention; water intake, serum glucose, total cholesterol, AST, and ALT values remained comparable between vehicle- and probiotic-treated groups. Notably, serum triglyceride levels were reduced in the Switch+EtOH-LrLl group compared to the Switch+EtOH-Veh. These findings suggest that the LrLl probiotic formulation can modulate ethanol consumption behavior and improve lipid metabolism, particularly in animals previously exposed to an HSB diet.

The probiotic blend influenced the restoration of the intestinal epithelium in ethanol-exposed animals, as evidenced by upregulation of *Muc2* and an increase in goblet-cell numbers in the Switch+EtOH-LrLl group. Several studies have shown that *L. lactis* NCDO 2118 and *L. rhamnosus* strains promote intestinal mucosal repair by enhancing *Muc2* transcription^23,24^. Upregulation of *Tjp-1* and *Cldn7* in the same group supports the capacity of these strains to reinforce barrier integrity^25^. These findings suggest that the probiotic blend strengthens the intestinal barrier, potentially preventing translocation of pathogens and endotoxins. In parallel, the LrLl blend reshaped the colonic microbiota. Whereas the relative abundances of phyla *Bacillota*, *Bacteroidota*, and *Proteobacteria* remained unchanged, the Switch+EtOH-Veh group showed an increase in Actinomycetota. This enrichment is consistent with studies associating elevated *Actinomycetota* with high ethanol intake and with gut and liver inflammation^6–26^. By restoring *Actinomycetota* abundance to baseline, the probiotic likely contributes to the decreased bacterial translocation and hepatic steatosis observed in the LrLl-treated animals.

The decrease in acetaldehyde in the Switch+EtOH-LrLl group, combined with histological evidence of hepatic recovery, indicates that *L. rhamnosus* and *L. lactis* contribute to the restoration of liver function. However, we did not directly quantify the acetaldehyde-converting activity of *Lacticaseibacillus rhamnosus* L156.4 or *Lactococcus lactis* NCDO 2118 in this study. Therefore, the contribution of bacterial acetaldehyde metabolism to the observed reductions in serum acetaldehyde and liver injury remains inferential and should be addressed in future work. Given the impact of intestinal and hepatic homeostasis on brain modulation^27^, we proceeded with behavioral analyses using the Open Field Test and Marble Burying Test. In these tests, exploratory and compulsive-like behaviors were attenuated in the LrLl groups, suggesting that the probiotic blend exerts effects on brain circuits involved in behavior modulation. At the molecular level, this behavioral attenuation corresponds with changes in the transcriptional regulation of dopaminergic signaling components within the striatum, a key region implicated in reward and addiction. Alterations in striatal *Drd2* signaling and transcriptional regulation have been associated with ethanol-seeking phenotypes^28–31^. A particularly striatal mechanism linking *Drd2* to ethanol preference involves the removal of extracellular dopamine from the synaptic cleft by dopamine transporters (DAT), a process that is highly sensitive to drugs of abuse^33–35^. Consistent with this, the *Slc6a3* transcripts (encoding DAT) were higher in Switch+EtOH-Veh than in Switch+EtOH-LrLl, indicating reduced DAT transcription in probiotic-treated ethanol-exposed mice. Extracellular dopamine homeostasis relies on two complementary pathways: rapid reuptake via DAT and slower degradation by catechol-O-methyltransferase (COMT), which together ensure sustained clearance supporting behavioral flexibility and reward sensitivity^36–38^. The concurrent upregulation of *Comt* and downregulation of *Slc6a3* in probiotic-treated animals, therefore, supports a shift toward enzymatic dopamine clearance as DAT transcripts decrease, aligning with the observed reduction in ethanol-seeking behavior. Taken together, these transcriptional shifts support probiotic-associated modulation of dopaminergic homeostasis within ethanol-exposed animals, rather than an ethanol-versus-non-ethanol effect on striatal *Drd2* transcript levels.

In this context, the probiotic blend should not be viewed only as reversing alcohol-driven molecular changes, but as modulating gut–brain physiological pathways that influence reward processing and ethanol reinforcement. Such modulation may occur in the absence of ethanol exposure yet becomes behaviorally relevant when animals engage in ethanol seeking and preference. Several studies suggest that *Gabbr* genes, which encode subunits of the GABA_B_ receptor, exhibit asymmetrical alterations in the brains of individuals with AUD, with *Gabbr1* mRNA showing more pronounced dysregulation^39,40^. The transcriptional increase of *Gabbr1* in the Switch+EtOH-LrLl group suggests that restoring this constitutive subunit may contribute to reducing dopaminergic hyperactivity in response to alcohol. This interpretation is reinforced by reports of molecular correlations between *Slc6a3* and *Gabbr1*, whose coordinated regulation appears to be influenced by *Lacticaseibacillus rhamnosus* in the brain, contributing to the adjustment of reward-related neural circuits^41,42^.

Although the specific role of *Lrrk2* in ethanol-related neuroadaptations remains unclear, the observed transcriptional regulation of this gene may reflect its involvement at the molecular interface between dopaminergic and immune signaling pathways^29,43–45^. Altered *Lrrk2* transcript levels, whether elevated or reduced, have been described across distinct phenotypes of alcohol intake and preference, often in association with changes in *Drd1* and *Drd2* transcription in striatal circuits of ethanol-exposed animals^28,29^. Moreover, the transcriptional regulation of *Lrrk2*, observed in parallel with adjustments in immune response gene transcripts in the Switch+EtOH-LrLl group, suggests that this gene may be responsive to diverse physiological stimuli or, alternatively, may participate in the modulation of synaptic and immunoadaptive processes involved in the consolidation of ethanol preference behaviors. *Lrrk2* transcriptional changes were accompanied by reduced mRNA levels of inflammatory mediators, including the nuclear transcription factor of activated T cells (*Nfatc1*), whose attenuation is associated with harmful feedback mechanisms that restrain proinflammatory interleukin activity^44,45^. Data obtained in the striatum of ethanol-exposed mice showing *Lrrk2* downregulation correlated with increased transcription of *Nfatc1*, *Il-1β*, *Il-6*, and *Tlr4*, suggesting that *Lrrk2* regulation contributes to an anti-inflammatory transcriptional profile in the nervous system^29,44–48^. Supporting this, evidence from murine models indicates that specific probiotic strains can modulate similar neuroimmune pathways. In particular, the regulatory activity of *L. rhamnosus* and *L. lactis* on the transcription of of *Il-1β*, *Il-6*, and *Tlr4* within brain reward circuits of ethanol-exposed mice has been associated with the behavioral modulation, including attenuation of compulsive and anxiety-like responses, which is consistent with the findings in the Switch+EtOH-LrLl group^49–51^. Furthermore, the downregulation of *Nos2* (iNOS) and *Cxcl2* (encoding a chemokine involved in neutrophil recruitment) was associated with reduced ethanol-induced cellular stress in probiotic-treated groups^51,52^.

The data presented here support our initial hypothesis that therapeutic intervention with the probiotic blend containing *L. rhamnosus* L156.4 and *L. lactis* NCDO 2118 can reduce high ethanol intake and preference in mice, while simultaneously restoring structural and functional integrity along the gut–brain axis. The safety of co-administering these strains supports their combined use and has been associated with improve immune and metabolic regulation. This is supported by the present findings and by studies showing beneficial effects when *Lactococcus lactis* and *Lacticaseibacillus* strains are administered together to mice exposed to a high-calorie diet for eight weeks, as well as when administered individually in alcohol preference paradigms^18,53^. Our findings indicate that the probiotic blend modulates dopaminergic signaling by balancing striatal *Drd2* and *Slc6a3* transcription in association with immune mediators, particularly within ethanol-exposed animals. This effect may be mediated by *Lrrk2*, which appears to influence *Drd1/Drd2* dynamics and is consistent with the transcriptional regulation of *Nfatc1* and several interleukins. Together, these observations support the hypothesis that the gut microbiota can modulate reward-related pathways through the regulation of dopaminergic gene transcription. Consistent with this idea, studies have shown that *Lacticaseibacillus rhamnosus* administration upregulates genes involved in reward processing, in parallel with changes in the abundance of gut microbial taxa such as *Bacillota* and *Bacteroidota*^40,41^. These findings suggest that specific bacterial taxa and their metabolites may help fine-tune dopaminergic tone, particularly during ethanol-induced neurochemical imbalance. One limitation of our study is that the animals were not pathogen-free and could exhibit microbiota-dependent outcomes based on previous microbiota composition, which may affect both treatment reproducibility and vulnerability to the addictive phenotype.

Exposure to a HSB protocol inherently alters the pool of available substrates and favors specific bacterial taxa. Although ethanol consumption was not the only variable in the experimental design, all groups received comparable diets over the same time interval, enabling microbiota comparisons among groups. That said, the focus of our study was the analysis of preference behavior driven by positive reinforcement transfer and compulsivity following withdrawal from the HSB diet, a phenomenon consistently reproduced in several of our previous studies. Our data demonstrate that ethanol consumption coupled with HSB diet withdrawal can induce dysbiosis within bacterial communities and may contribute to the development of ethanol dependence-like behavior, regardless of the initial microbial ecosystem. Because 16 S rRNA sequencing provides relative abundance data and lacks species-level resolution, integrating metagenomic and/or metabolomic analyses would be valuable to further elucidate the metabolic consequences of the observed microbial shifts. In particular, mapping microbial composition and metabolite profiles onto behavioral data may help identify metabolic pathways most relevant to alcohol-related behaviors, potentially revealing novel therapeutic targets to reduce or prevent alcohol consumption. Future complementary studies in females are planned, with appropriate design considerations to evaluate generalization and potential sex-specific effects^54,55^.

## Methods

### Animals

Twenty-five conventional male C57BL/6 mice (6 weeks old), with an average body weight of 20.35 ± 2.64 g, obtained from the Universidade Federal de Minas Gerais (UFMG) were housed individually in micro-isolators for 12 weeks under controlled conditions with a 12-hour light/dark cycle (lights on at 7:00 a.m. and off at 7:00 p.m.). Only male mice were used in this study to avoid potential confounding effects of estrous cycle–related hormonal fluctuations on ethanol intake and corticostriatal circuit function. All experimental procedures were approved by the UFMG Animal Ethics Committee (CEUA protocol 243/2022). All methods were carried out in accordance with guidelines and regulations for the care and use of laboratory animals in Brazil. This study was conducted and reported in accordance with the ARRIVE guidelines. Animal welfare was prioritized throughout the study.

### Experimental design

The study design (Fig. 1a) was adapted from an animal model previously developed by our research group to investigate the neuronal circuits underlying high ethanol consumption and preference^18,22^. This framework has been shown by our group to reliably produce high voluntary ethanol intake and preference under the free-choice paradigm^5–7^. After one 1-week of acclimation, conventional C57BL/6 mice (*n* = 20) were fed a High Sugar and Butter (HSB) diet for 8 weeks; this dietary protocol was designated T1. During T2 (weeks 9–10), the HSB diet was replaced with the the standard AIN93G diet, and the mice were placed in a two-bottle free choice paradigm between water and 10% (v/v) ethanol solution, forming the Switch+EtOH group (*n* = 10), wheareas animals not exposed to ethanol formed the Switch group (*n* = 10). Switch animals had a single water bottle, whereas Switch+EtOH animals had two bottles (water vs. 10% (v/v) ethanol solution). In T3, beginning in week 11, a probiotic (LrLl) was introduced alongside the free-choice paradigm, subdividing the previous groups into Switch-Veh (*n* = 5), Switch-LrLl (*n* = 5), Switch+EtOH-Veh (*n* = 5), and Switch+EtOH-LrLl (*n* = 5). Animals had access to the diet throughout T1, T2, and T3, whereas ethanol exposure occurred from T2 to T3, and probiotic administration was restricted to T3. During T2, animals had ad libitum access to diet, ethanol, and water for 24 h. In contrast, during T3, all liquid solutions were withdrawn during the 3.5-hour probiotic exposure period, after which the standard access to diet and solutions was reestablished. Therefore, during T3, ethanol and water bottles were available for 20.5 h/day, and this access time was used for intake normalization when reporting consumption rates (g/kg/h). The experiment concluded at week 12. Bottles containing water or ethanol solutions were weighed and replaced daily at 8:00 a.m. Probiotic- or medium-containing solutions were administered during the light cycle, between 2:00 p.m. and 5:30 p.m. Water, ethanol solution, and treatment intake were estimated by weighting the bottle and calculating the difference between initial and final values recorded after each exposure period. A naïve group maintained exclusively on the AIN93G diet and water throughout the experiment provided baseline values for each experimental variable (e.g., ethanol intake, behavioral scores, metabolic states or probiotic supplementation). The mean value of the naïve group was calculated and used as a normalization factor: values from the ethanol and probiotic groups were expressed relative to this baseline (e.g., % of naïve mean). This approach allows comparison across groups while controlling for inter-animal variability and day-to-day experimental fluctuations. At the end of the experimental period, all animals were euthanized under deep anesthesia using a ketamine (100 mg/kg) and xylazine (10 mg/kg) solution administered intraperitoneally.

### Probiotic blend

The probiotic blend was administered orally to the mice via drinking bottles for 3.5 h daily (2:00–5:30 PM), serving as the sole liquid source, with solid diet available ad libitum. The strains were *Lacticaseibacillus rhamnosus* L156.4 and *Lactococcus lactis* NCDO 2118. *L*, cultured anaerobically for 18 h (*L. lactis* in MRS at 30 °C; *L. rhamnosus* in MRS + Tween 80 at 37 °C), then subcultured (3–5 h) to reach ~ 10⁹ CFU/mL and mixed 1:1. The vehicle control consisted of sterile, pH-matched MRS broth prepared without bacteria (a nutrient medium containing peptone/extracts, glucose, acetate/citrate buffers, and mineral salts), ensuring any nutrient contribution was matched across groups.

### Ethanol intake and preference

Water and 10% (v/v) ethanol solution consumption were quantified during each 20.5 h access period by weighing the bottles before and after access. Intake was calculated as the difference in bottle weight (g). Because bottle weight difference was the primary measured variable in this experiment, these gravimetric values were used as the basis for all subsequent calculations. After normalization to body weight, ethanol intake was additionally expressed as pure ethanol intake (g/kg/day) in the manuscript and as 10% ethanol solution intake (g/kg/day and mL/kg/day) in the supplementary material to facilitate comparison with the literature. For these conversions, the mass of the consumed 10% (v/v) ethanol solution was converted to volume assuming that 1 g of solution corresponded to approximately 1.014 mL, and pure ethanol mass was estimated using ethanol density (0.789 g/mL). The ethanol solution was replaced after each weighing to limit evaporative loss. Ethanol preference was calculated as the percentage of total fluid intake derived from the ethanol bottle (ethanol solution intake / [ethanol solution intake + water intake] × 100). A significant preference was recorded when ethanol accounted for > 50.1% of total fluid intake (*p* < 0.05).

### Serum biochemical analyses

At the end of the experiment, whole blood was collected by cardiac puncture (terminal collection), allowed to clot, and centrifuged to obtain serum. Serum total cholesterol, glucose, triglycerides, aspartate aminotransferase (AST), and alanine aminotransferase (ALT) were measured using enzymatic colorimetric assay kits (Bioclin, Brazil) in accordance with the manufacturer’s instructions.

### Colon histopathology

Colon tissue was collected, rinsed in 0.1 M PBS (pH 7.2), rolled, and fixed in 4% buffered formaldehyde. Samples were paraffin-embedded, sectioned at 4 μm, mounted on glass slides, and stained with Hematoxylin-Eosin (HE). Histological scoring evaluating seven parameters: inflammation, edema, hyperemia/vasodilation, cellular infiltration, superficial epithelial injury, crypt involvement, and mucus presence, each graded from 0 (absent) to 3 (marked). For mucin assessment, adjacent sections were stained with Periodic acid–Schiff (PAS), and goblet cells within the Paneth-cell compartment were enumerated. Ten non-overlapping fields per sample were photographed at 40× magnification, and goblet cells were counted manually were counted manually using ImageJ (Cell Counter plugin). Results are expressed as cells per high-power field.

### Transcriptional quantification (qPCR)

Total RNA was isolated from colon and striatum tissues using the reliapprep™ RNA Tissue Miniprep System (PROMEGA, USA). Concentration and purity were assessed with a Denovix ds-11 spectrophotometer. First-strand cDNA synthesis was performed from 500 ng of total RNA, 10 µM oligo(dT)_20_ primers (Phoneutria Biotecnologia, Brazil). Transcriptional regulation was evaluated by RT-qPCR on the CFX 96TM Real-Time System (Biorad, USA), using SYBR Green PCR Master Mix (Applied Biosystems, USA). Primer sequences are listed in supplementary Table 1. *Gapdh* was used as the endogenous reference gene. Relative transcript levels were calculated with the comparative 2^–ΔΔCt^ method, taking the mean ΔCt of the control group as the calibrator.

### Absolute qPCR of colon bacterial groups

Absolute bacterial counts were obtained by quantitative real-time PCR (qPCR). Total DNA was extracted from 0.15 g of intestinal content with the QIAamp DNA Stool Mini Kit (QIAGEN, Brazil). Duplicate 20 µL reactions were run on a CFX96™ system (Bio-Rad, USA) containing 10 µL SYBR Green Master Mix (Kapa Biosystems, Brazil), 0.4 µL of each primer (10 µM), 1 µL template DNA (12.5 ng), and nuclease-free water; no-template controls received water instead of DNA. The cycling profile was 95 °C for 10 min, then 40 cycles of 95 °C for 15 s and 60 °C for 1 min, with fluorescence read at the end of each annealing/extension step. Copy numbers were calculated from standard curves generated using 10-fold serial dilutions of genomic DNA from reference strains, corrected for the 16 S rRNA operon count of each taxon using rrnDB v5.9, and expressed as a percentage of the total bacterial load for each group. Primer sequences and rrn copy numbers are listed in supplementary Table 2.

### Liver histopathology

Liver tissue was fixed in 10% neutral-buffered formalin, paraffin-embedded, sectioned at 5 μm, stained with H&E, and digitized on a Pannoramic slide scanner (3DHISTECH, Hungary). Steatosis was graded 0–4 according to the proportion of fat-containing hepatocytes per field (< 5%, 5–25%, 26–50%, 51–75%, > 75%). Lobular inflammation was graded (score 0–3) based on the average number of inflammatory foci (defined as clusters of infiltrating mononuclear cells) at ×200 magnification per area (cm²). Two independent investigators blinded to the samples evaluated the liver histological slides to minimize interobserver variation.

### Serum acetaldehyde quantification

Serum acetaldehyde was measured using the Acetaldehyde Assay Kit (Sigma-Aldrich, USA), following the manufacturer’s instructions. After serum separation, samples were aliquoted and stored at − 80 °C until analysis. Acetaldehyde was quantified after short-term storage, and all samples were assayed in the same analytical batch to minimize volatilization/degradation and inter-assay variability. Briefly, samples were diluted in a ratio of 1:10, and aldehyde dehydrogenase catalyzed acetaldehyde oxidation, generating NADH, which reduced a fluorogenic probe. After a 30-minute incubation at room temperature, fluorescence was read in a microplate reader at λex = 530 nm/λem = 585 nm. Samples and standards were prepared in duplicate. Concentrations were calculated from a standard curve and expressed in µM.

### Bacterial translocation

Total DNA was extracted from liver specimens collected at the end of T3 using the DNeasy Blood & Tissue Kit (QIAGEN, Germany). Bacterial translocation was assessed by absolute qPCR on a CFX96™ Real-Time PCR System (Bio-Rad, USA) with SYBR Green Master Mix (Kapa Biosystems, USA) and the universal 16 S rRNA primers 926 F (5′-AAACTCAAAKGAATTGACGG-3′) and 1062R (5′-CTCACRRCACGAGCTG-3′). Cell equivalents (bacteria µg⁻¹ liver DNA) were derived from Ct values plotted against a 10-fold genomic-DNA standard curve and normalized assuming 4.9 16 S operons per genome (rrnDB v5.9)^56–60^.

### Behavioral tests

Obsessive-compulsive–like behavior was assessed three days before the end of T3 using the marble-burying test. Each mouse was placed individually in a standard cage containing 5 cm of sawdust bedding, in which 18 glass marbles were arranged in three rows of six. After 10 min, the number of marbles buried to at least two-thirds of their depth was recorded^61^. Exploratory activity and locomotion were evaluated two days before the end of T3 using the open-field test. Animals were placed in the center of a square arena (40 × 40 × 40 cm; opaque walls) and video-tracked for 5 min with an overhead camera. Recordings were analyzed offline in EthoVision XT (Noldus Information Technology, Netherlands) to obtain immobility time, number of rotations, central-zone entries, and total distance traveled^62^.

### Statistical analyses

Normality was assessed with the D’Agostino-Pearson and Shapiro-Wilk tests. Parametric data were analyzed with ordinary two-way ANOVA or two-way repeated measures ANOVA, followed, when appropriate, by Sidak or Tukey post hoc tests. Independent two-group comparisons used the unpaired Student t test, and ethanol preference was evaluated with a one-sample t test against the theoretical value of 50.1%. Non-parametric data were analyzed using the Kruskal-Wallis test and Dunn multiple comparisons. Correlations were examined using Pearson’s r and simple linear regression. All analyses were performed in GraphPad Prism version 9.02 (GraphPad Software, USA). Data are reported as mean ± SEM, and significance was set at *p* < 0.05.

## Supplementary Information

Below is the link to the electronic supplementary material.

Supplementary Material 1

## Data Availability

The datasets used and/or analyzed during the current study are available from the corresponding author on reasonable requests.
