# Supplementary material for: Effects of Lacticaseibacillus rhamnosus L156.4 and Lactococcus lactis NCDO 2118 strains on reducing alcohol intake and preference in an animal model of high alcohol consumption and preference
Source: Sci Rep. 2026 Apr 14;16:17307. doi: 10.1038/s41598-026-48677-y (PMC13234130; doi:10.1038/s41598-026-48677-y)
Supplement: Supplementary file 1 — Supplementary Material 1 [file 41598_2026_48677_MOESM1_ESM.docx]

**SUPPLEMENTARY INFORMATION**


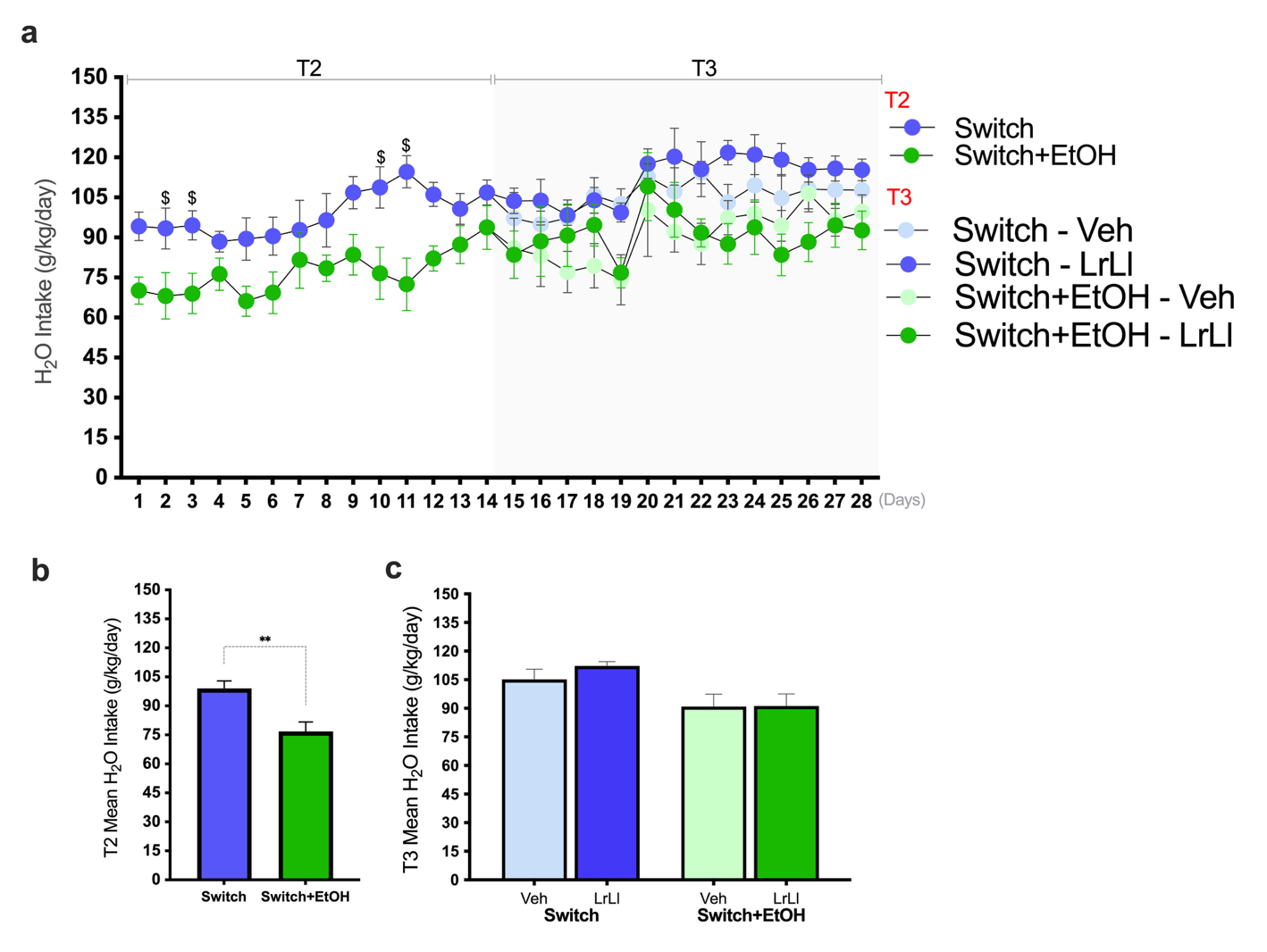


**Supplementary Figure S1**: **Time-course of water intake.** (a) Daily H_2_O intake (g/kg/day). (b) Mean H_2_O intake during T2 (g/kg/day). (c) Mean H_2_O intake during T3 (g/kg/day). Data were analyzed by (a) two-way repeated-measures ANOVA with Sidak’s post hoc test, (b) unpaired t-test, and (c) one-way ANOVA. **p <0.01; ^$^p < 0.05. In this experiment, one day corresponded to 20.5 h of access.


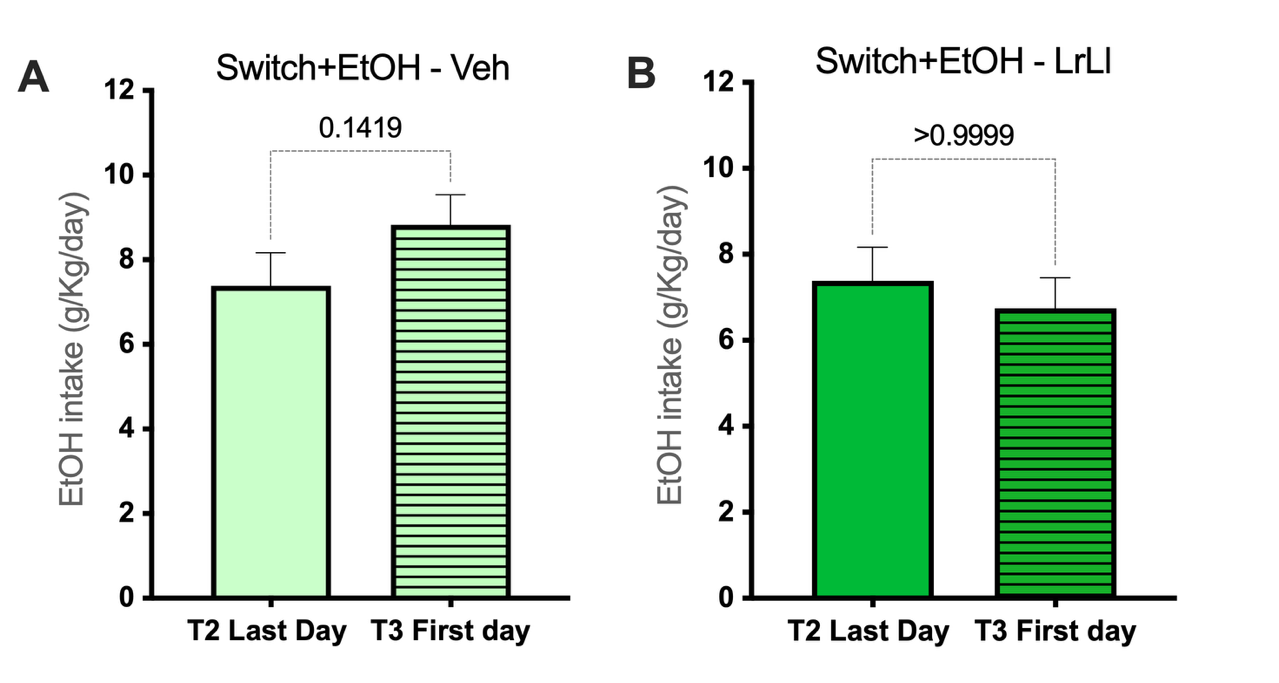


**Supplementary Figure S2:** Statistical comparison of ethanol intake between the last day of T2 and the first day of T3. Despite the apparent increase in the Vehicle group and decrease in the LrLI group at this transition point, no significant differences were detected (Switch+EtOH–Veh, *p* = 0.1419; Switch+EtOH–LrLI, *p* > 0.9999).


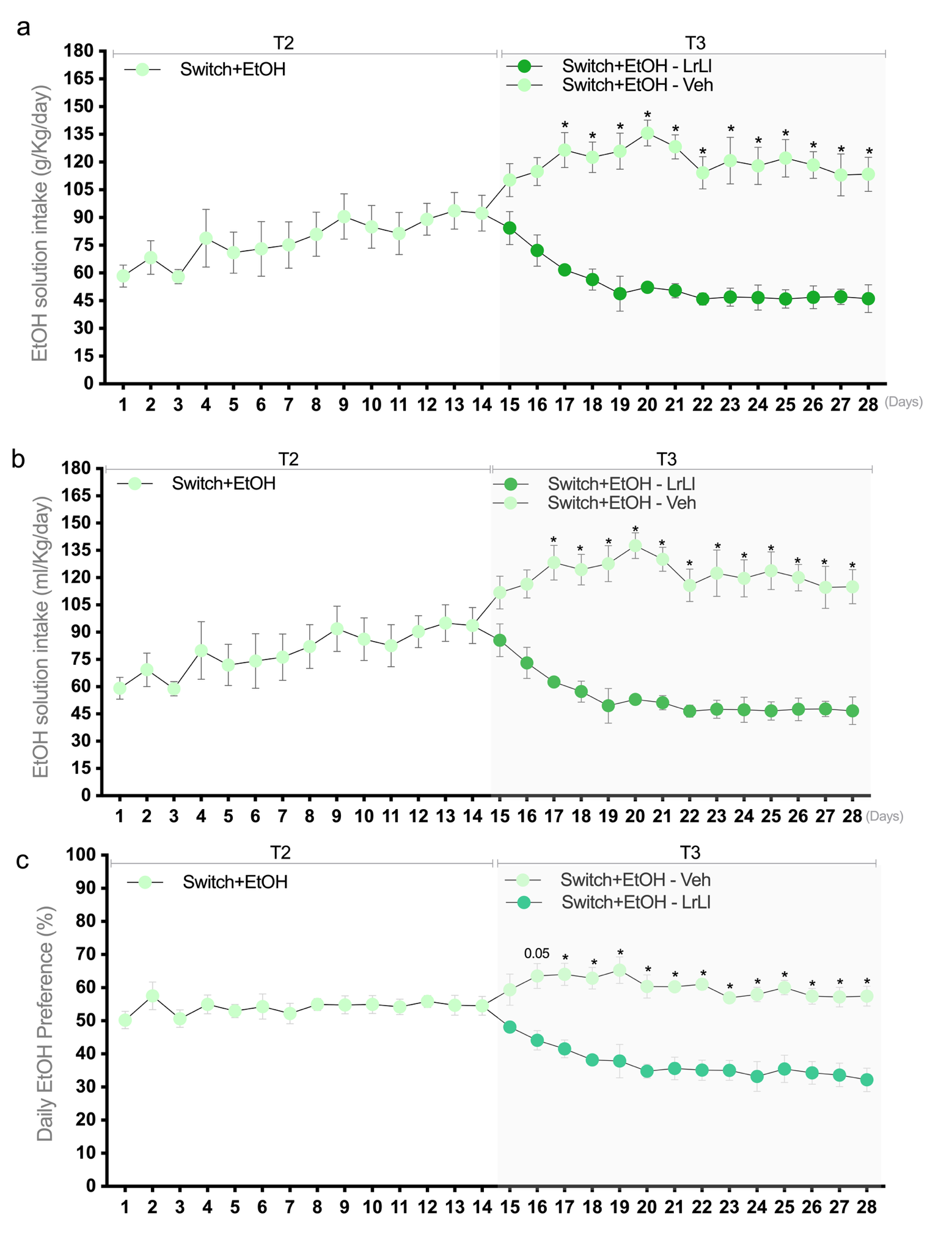


**Supplementary Figure S3**: Voluntary ethanol intake and daily preference during phases T2 and T3. (a) 10% (v/v) ethanol solution intake (g/kg/day). (b) 10% (v/v) ethanol solution intake (mL/kg/day). (c) Daily ethanol preference (%) averaged by group. Data were analyzed using two-way repeated-measures ANOVA followed by Sidak’s multiple-comparisons test. *p < 0.05. In this experiment, one day corresponded to 20.5 h of access.


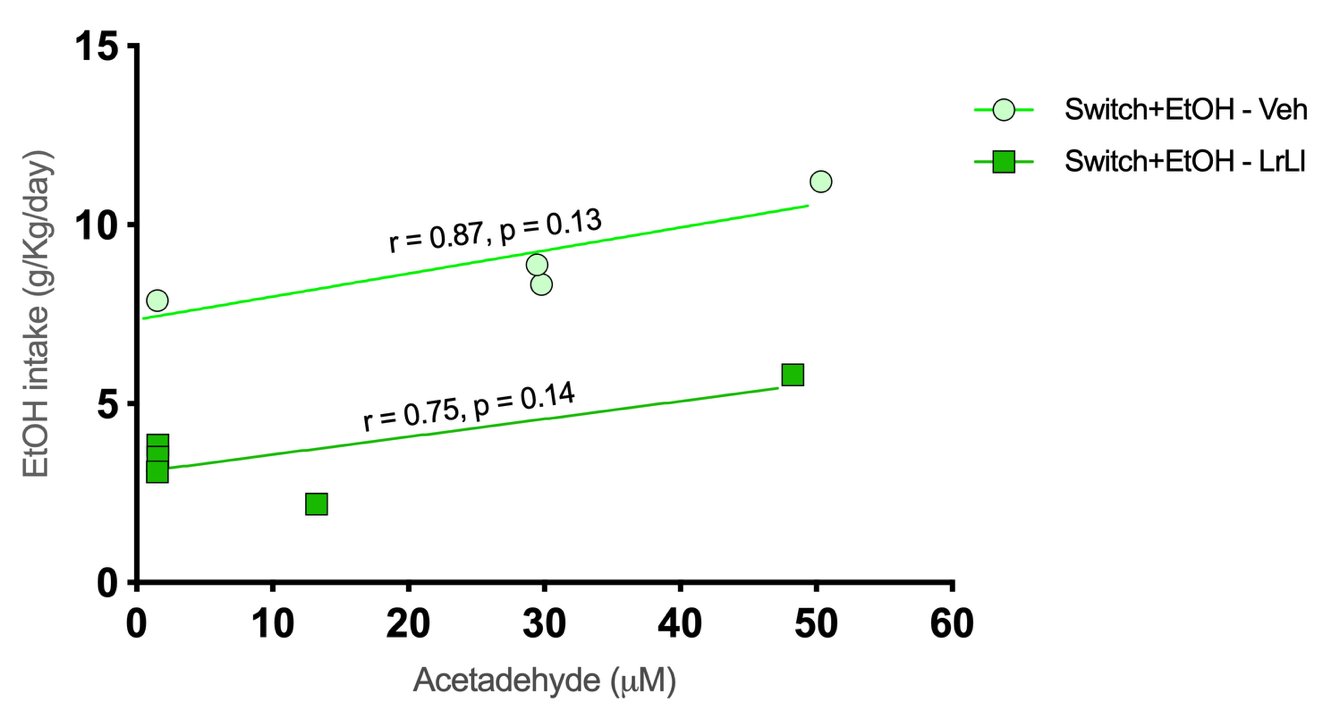


**Supplementary Figure S4:** Correlation between serum acetaldehyde levels and ethanol intake (g/kg/h) on the last day of the experiment. A positive trend was observed between higher alcohol consumption and acetaldehyde levels in both groups (Switch+EtOH–Veh, r = 0.87, *p* = 0.13; Switch+EtOH–LrLI, r = 0.75, *p* = 0.14). Given the small sample size (*n* = 5 per group after subdivision), statistical power was limited, and correlations did not reach conventional significance. However, the effect direction was supported by simple linear regression showing positive slopes in both groups (Switch+EtOH–Veh, *Y* = 0.06439**X* + 7.348, 95% CI of slope −0.04534 to 0.1741, *p* = 0.1275; Switch+EtOH–LrLI, *Y* = 0.04978**X* + 3.078, 95% CI of slope -0.03004 to 0.1296, *p* = 0.1414).


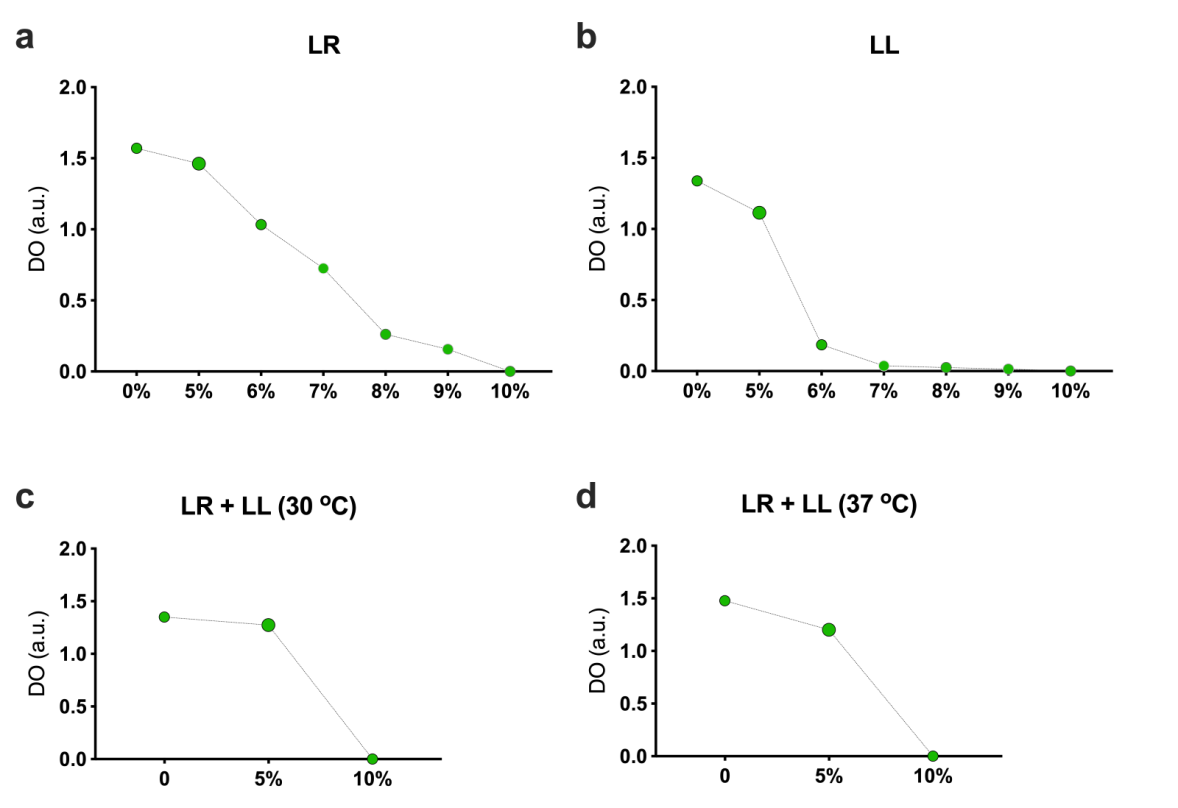


**Supplementary Figure S5**: **Ethanol tolerance of** Lacticaseibacillus rhamnosus L156.4 (Lr) and Lactococcus lactis NCDO 2118 (Ll)**.** (a) LR cultivated in strain-specific medium containing 0–10 % (v/v) ethanol. (b) LL cultivated with 0–10% ethanol. (c) LR + LL co-culture incubated at 30 °C with 0, 5, and 10% ethanol. (d) LR + LL co-culture incubated at 37 °C with 0, 5, and 10 % ethanol. Optical density (OD, arbitrary units) was normalized to the maximum OD for each strain (1.6 for LR, 1.4 for LL). Both strains maintained growth up to 5% ethanol, whereas 10% completely inhibited proliferation under all conditions. Data represent the mean of independent experiments.

**Supplementary Table S1:** Primers (5'→3') used for RT-qPCR

| Genes | Forward (5’- 3’) | Reverse (5’- 3’) |
| --- | --- | --- |
| *Gapdh* | AGGAGCGAGACCCCACTAAC | GTGGTTCACACCCATCACAA |
| *Drd1* | GAGTCGGGGAGTGGTCT | CAATCTCAGTCACTTTTCGGG |
| *Drd2* | GCCAACCTGAAGACACCACT | CTTGACAGCATCTCCATTTCC |
| *Gabbr1* | TCCACCAACAACAATGAGGA | GATGGCGCAGTTCAGAGAC |
| *Gabbr2* | GAGGACATCAACTCCCCAGA | CTGGCTGTAGGGCTGACAC |
| *Lrrk2* | TTCCCCACCAATGAAAACAT | AAGGCTGCGTTCTCAGGATA |
| *Comt* | GTGCTTTGAAGATGCCGGAG | GTGTGCTTTGCATTTAGGACA |
| *Slc6a* | TTCCGAGAGAAACTGGCCTA | TGTGAAGAGCAGGTGTCCAG |
| *Snca* | CACTGGCTTTGTCAAGAAGGAC | CATAAGCCTCACTGCCAGGAT |
| *Nfat* | CAGTGTGACCCAAGATACCTGG | TCGAGACTTGATAGGGACCCC |
| *Bdnf* | TGGCTGACACTTTTGAGCAC | AAGTGTACAAGTCCGCGTCC |
| *Tlr4* | AGTAGCACTGACACCTTCCTT | GCCTTAGCCTCTTCTCCTTCA |
| *Il-1B* | CACTCATTGTGGCTCTGGAGAA | CCACGGGAAAGACACAGGTA |
| *Il-6* | CTCTGGGAAATCGTGGAAATG | AAGTGCATCATCGTTGTTCATA |
| *iNos* | AGCACTTTGGTGACCACCAGGA | AGCTAAGTATTAGGAGCATGTG |
| *Cxcl2* | AGGTCCCTGTCATGCTTCTG | TCTGGACCCATTCCTTCCTTG |
| *Ppia* | AATGCTGGACCAAACACAAA | GTGGTTCACACCCATCACAA |
| *Muc2* | ACTCTCCACACCCAGCATCATC | GTGTCTCCGTATGTGCCGTTGT |
| *Cldn7* | GGAGACGACAAAGTGAAGAAG | AAAAGTCTGTGACAATCTGAGG |
| *Ocl* | GGCGGATATACAGACCCAAGA | GATAATCATGAACCCCAGGAC |
| *Zo1* | CCACCGGAGTCTGCCATTACAC | GGGTGGGCGCGGACTATCTGA |
| *Tpj-1* | GTCCAGAATCTCGGAAAAGTGC | CTTTCAGCGCACCATACCAACC |

Reference gene: Glyceraldehyde-3-phosphate dehydrogenase (*Gapdh*). Target genes: dopamine receptors 1 (*Drd1*) and 2 (*Drd2*); GABA receptors B (*Gabbr1*), GABA 2 (*Gabbr2*), Leucine-rich repeat kinase 2 (*Lrrk2*); dopamine transporter (*Slc6a*); Catechol-O-Methyl Transferase (*Comt*); Alpha-Synuclein Gene (*Snca*); Nuclear Factor of Activated T cells (*Nfat*);); Toll-Like Receptor 4 (*Tlr4*); Interleukin 1 Beta (*Il-1B*); Interleukin 6 (*Il-6*);  Inducible nitric oxide synthase (iNos); C-X-C motif chemokine ligand 2 (*Cxcl2*); Peptidylprolyl isomerase A (*Ppia*); Mucin 2 (Muc2); Claudin 7 (*Cldn7*); Occludin (*Ocld*); Zonulin 1 (*Zo1*) and Tight Junction Protein 1 (*Tpj*-*1*).

**Supplementary Table S2:** Primers (5'→3') used for Absolute qPCR of colon bacterial groups

| Alvo | Sequência (5’ – 3’) | Amplicon | TM |
| --- | --- | --- | --- |
| *Eubacteria* | AAACTCAAAKGAATTGACGG  CTCACRRCACGAGCTGAC | 180 | 60 |
| *Bacillota* | GGAGYATGTGGTTTAATTCGAAGC  AGCTGACGACAACCATGCAC | 126 | 60 |
| *Bacteroidota* | CRAACAGGATTAGATACCCT GGTAAGGTTCCTCGCGTAT | 240 | 60 |
| *Proteobacteria* | TCGTCAGCTCGTGTYGTGA CGTAAGGGCCATGATG | 170 | 60 |
| *Actinomycetota* | TACGGCCGCAAGGCTA  TCRTCCCCACCTTCCTCCG | 170 | 60 |
| *Lacticaseibacillus rhamnosus* | GGCATGCCGACTACGTTAAG  GTTCACGTGTTTGTGGCATT | 158 | 60 |
| *Lactococcus lactis* | TGGCTCAGGACGAACGCT  CCTCTCAGG TCGGCTATGTAA | 107 | 60 |

Primer sequences used for quantitative PCR (qPCR) targeting specific bacterial groups. Primer pairs were designed to amplify regions of the 16S rRNA gene, enabling absolute quantification of total bacteria (Eubacteria), and specific phyla or genera including *Bacillota*, *Actinomycetota*, *Lacticaseibacillus rhamnosus*, and *Lactococcus lactis*. Amplicon size (in base pairs), annealing temperature (Tm, in °C), and reference sources are indicated.

**Supplementary Table S3:** Experimental diet compositions (g/kg diet).

| **Ingredient** | AIN93G [1] | HSB [2] | Role/Function |
| --- | --- | --- | --- |
| Casein | 200.0 | 200.0 | Protein source |
| Corn starch | 397.5 | 208.6 | Carbohydrate |
| Sugar | 100.0 | 232.0 | Carbohydrate |
| Destrinized starch | 132.0 | 0.0 | Carbohydrate |
| Commercial butter | 0.0 | 189.0 | Saturated-fat source |
| Soybean oil | 70.0 | 70.0 | Essential fatty acids |
| Cellulose | 50.0 | 50.0 | Fiber |
| Mineral mix (AIN-93G) | 35.0 | 35.0 | Minerals |
| Vitamin mix (AIN-93G) | 10.0 | 10.0 | Vitamins |
| L-Cystine | 3.0 | 3.0 | Sulfur amino acid |
| Choline bitartrate | 2.5 | 2.5 | Methyl donor |
| BHT | 0.014 | 0.014 | Antioxidant |
| **Total mass (g)** | 1000 | 1000 |  |
| **Energy density (Kcal/g)** | 3.9 | 4.9 |  |

**HSB** - High Sugar and Butter; **AIN93G** - American Institute of Nutrition 1993 Growth

**REFERENCES**

1. Reeves, P.G., F.H. Nielsen, and G.C. Fahey, *AIN-93 purified diets for laboratory rodents: final report of the American Institute of Nutrition ad hoc writing committee on the reformulation of the AIN-76A rodent diet.* J Nutr, 1993. **123**(11): p. 1939-51. DOI: [10.1093/jn/123.11.1939](https://doi.org/10.1093/jn/123.11.1939)

2. Maioli, T.U., et al., *High sugar and butter (HSB) diet induces obesity and metabolic syndrome with decrease in regulatory T cells in adipose tissue of mice.* Inflamm Res, 2016. **65**(2): p. 169-78. DOI: [10.1007/s00011-015-0902-1](https://doi.org/10.1007/s00011-015-0902-1)

**Supplementary Table S4**: Quantification assays used in the study.

| ***Experiment*** | ***Kit especification*** | ***Mark*** | ***Reference*** |
| --- | --- | --- | --- |
| *Serum Quantification* | *Acetaldehyde Assay Kit* | Sigma-Aldrich, USA | Cat. No **MAK321.** |
| *Serum Biochemical Analyses* | *Transaminase ALT kinetics Kit* | Bioclin, Brazil | Cat. No **K049-6.** |
| *Serum Biochemical Analyses* | *Glucose measuring strips* | On Call Plus II, USA | Cat. No **80560310** |
| *Serum Biochemical Analyses* | *Transaminase AST kinetics Kit* | Bioclin, Brazil | Cat. No **K048-6.** |
| *Extraction of intestinal bacterial genomic DNA* | *QIAamp DNA Stool Mini Kit* | QIAGEN, Brazi | Cat. No. **51504**. |
| *Purification total DNA (translocation bacterial)* | *DNeasy Blood & Tissue Kit* | QIAGEN, Germany | Cat. No. **69504**. |
| *Total RNA extraction RT-PCR/cDNA synthesis* | *Reliapprep™ RNA Tissue Miniprep* | PROMEGA, USA | Cat. No. **Z6111.** |

**REFERENCES**

1 Acetaldehyde Assay Kit —
 https://www.sigmaaldrich.com/

2. ALT and AST Kinetic Kit —
https://quibasa.bioclin.com.br

3.Glucose Measuring Strips — https://www.gimaitaly.com/DocumentiGIMA/Manuali/EN/M23914EN.pdf

4.QIAamp DNA Stool Mini Kit — https://www.qiagen.com/br/shop/sample-technologies/dna/

5.DNeasy Blood & Tissue Kit — https://www.qiagen.com/br/shop/sample-technologies/dna/

6.ReliaPrep™ RNA Tissue — https://www.promega.com.br/
